# Cranial sutures work collectively to distribute strain throughout the reptile skull

**DOI:** 10.1098/rsif.2013.0442

**Published:** 2013-09-06

**Authors:** Neil Curtis, M. E. H. Jones, S. E. Evans, P. O'Higgins, M. J. Fagan

**Affiliations:** 1 Medical and Biological Engineering Research Group, School of Engineering, University of Hull, Hull HU6 7RX, UK; 2 Research Department of Cell and Developmental Biology, University College London, Gower Street, London WCIE 6BT, UK; 3 Centre for Anatomical and Human Sciences, Hull York Medical School, University of York, York YO10 5DD, UK

**Keywords:** cranial suture, multibody dynamics analysis, *Sphenodon*, finite-element analysis

## Abstract

The skull is composed of many bones that come together at sutures. These sutures are important sites of growth, and as growth ceases some become fused while others remain patent. Their mechanical behaviour and how they interact with changing form and loadings to ensure balanced craniofacial development is still poorly understood. Early suture fusion often leads to disfiguring syndromes, thus is it imperative that we understand the function of sutures more clearly. By applying advanced engineering modelling techniques, we reveal for the first time that patent sutures generate a more widely distributed, high level of strain throughout the reptile skull. Without patent sutures, large regions of the skull are only subjected to infrequent low-level strains that could weaken the bone and result in abnormal development. Sutures are therefore not only sites of bone growth, but could also be essential for the modulation of strains necessary for normal growth and development in reptiles.

## 1. Introduction

Skulls are made up of many bones that are connected by fibrocellular joints at sutures [1–6]. While the term ‘suture’ is sometimes used to refer solely to the soft tissue component of the joint [7], we use this term to include both the soft tissue component and the bone at the suture edges [8]. Sutures are sites of appositional bone growth [4,6,9–12] and are crucial in the developing skull, where their premature fusion can lead to abnormal growth [13–18]. As skulls mature, the sutures may become fused yet some remain patent throughout life [1,19], suggesting that they have an additional role. In some non-mammalian tetrapods, the joint between bones at patent sutures can be so large and flexible that the suture contributes to movements within the skull [20,21]. Nonetheless, more generally, the retention of patent sutures in mature skulls is thought to be related to stress transfer and/or stress dampening [5,22,23]. However, although the relative importance of each of these roles continues to be debated, their possible interactions merit more serious consideration. Thus, during growth patent sutures contribute not only to bone apposition but, given their patency and physical properties, must inevitably also impact on skull mechanics as they do in adults. In turn, the contribution of sutures to load transfer and to stress and strain modification within the skull [24–27] is likely to impact on cranial bone growth [28].

Virtual computational techniques such as finite-element analysis (FEA) are ideally suited to investigating the impact of patent sutures on skull stresses and strains. The established way to measure strains experimentally is with strain gauges fixed to the surface of bones [29–35]. Local strain at these specific locations is returned, but inferring either strain over the whole skull or the function of patent sutures is problematic. FEA allows stresses and strains to be predicted for the entire structure [36–44], and anatomical features to be controlled so that the influence of patent or fused sutures can be explored [45–51]. Moazen *et al*. [24] carried out such an analysis on a lizard skull, where specific sutures were modelled as patent within the computer model and their impact on local and global strains assessed. This study revealed that patent sutures modified strains over the skull compared with fused sutures, and that whereas strains decreased in some areas of the skull, they increased in others. However, Wang *et al*. [52] concluded that patent sutures have little effect on skull strains in primates, and that they are perhaps less important mechanically than in animals with more patent sutures or a greater suture to bone volume, such as lizards and alligators [24,53]. Such studies combined with experimental data provide important information on suture form and function [3,23,29,30,32,54], but for a full overview of the impact of patent and fused sutures on load transfer within the skull more comprehensive analyses are necessary. The skull experiences loads of varying location, direction and magnitude during normal everyday activities such as feeding, and the same is true for sutures. To appreciate fully the function or impact of sutures on skull stresses and strains, a range of loading regimens should be investigated. However, this was not done in previous studies.

Past work has suggested that: patent sutures do not affect strain distributions [52]; patent sutures act as strain sinks and reduce strains [22,23]; sutures both reduce and elevate strains [24] and that patent sutures help modulate strains throughout the skull [30,55,56]. Here, we investigate the impact of experimental, *in silico*, fusion of sutures on strain magnitudes and distributions within the skull of the reptile *Sphenodon* by testing the following hypotheses.

**Hypothesis 1:** sutures have no impact on strain distribution and magnitude in the skull.

**Hypothesis 2:** patent sutures reduce the mean strain across the skull.

**Hypothesis 3:** patent sutures lead to more uniform strain distributions in the skull.

To do this, we combine two computational techniques, multibody dynamics analysis (MDA) and FEA in the reptile *Sphenodon*, first to predict 15 separate biting loading regimens, and then to analyse the structural performance of the skull under these. The performance of the skull under many different loading regimens is important because the skull will deform differently dependent upon the loading position and magnitude. This is a consideration most other studies do not take into account.

**Figure 1.**
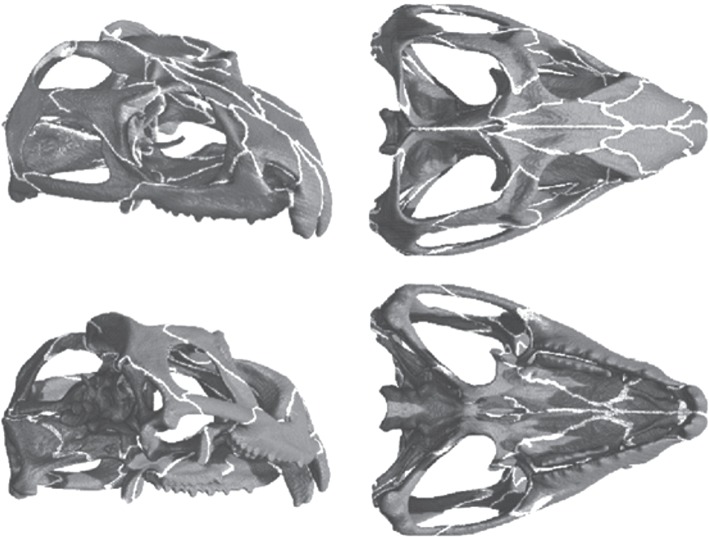
The skull model of the reptile *Sphenodon*. Grey regions of the skull represent bone material and white regions represent suture material.

There are over 100 sutural joints in the skull of *Sphenodon* [3], and all patent sutures were carefully incorporated into the model (figure 1). This level of suture modelling has not been carried out before. The deformation of each individual suture will impact on all other sutures, thus excluding one or more sutures from the model may affect the deformations of both the bones and the other sutures. Understanding the role of sutures with respect to load transfer in the skull of *Sphenodon*, where sutural anatomy is complex, can provide important information on general skull mechanics. In addition, identifying the overall contribution of patent sutures to load transfer through the skull may improve our understanding of medical conditions such as craniosynostosis, where skull growth is abnormal due to early suture fusion.

## 2. Material and methods

### 2.1. Multibody dynamics analysis

**Figure 2.**
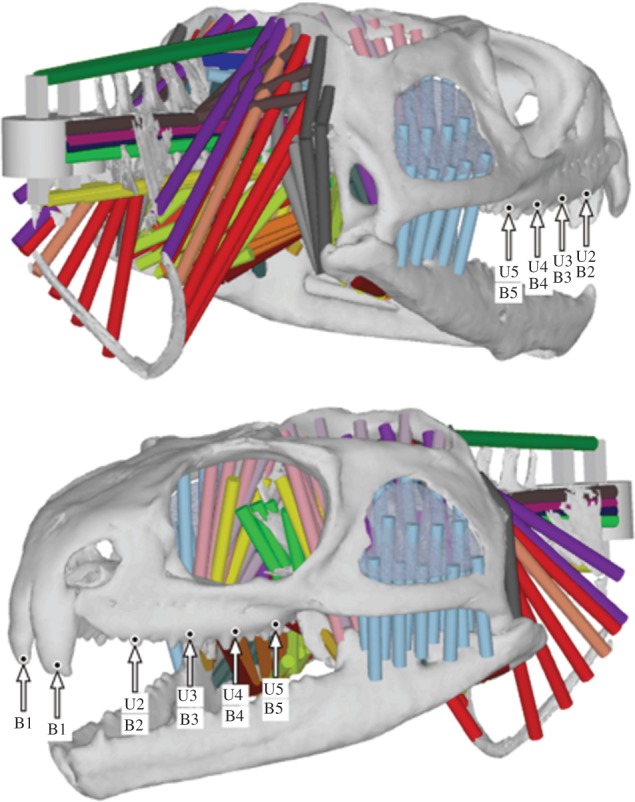
The MDA model highlighting bite location and type. U, unilateral bite; B, bilateral bite. Two ripping bites were also simulated at B2.

Detailed descriptions of the MDA model development have been presented elsewhere [57–61]. Briefly, the cranium and lower jaws (left and right parts) of a *Sphenodon* skull (specimen ID: LDUCZ x036) were scanned in-house by micro-computed tomography (micro-CT), and three-dimensional geometries were constructed using AMIRA image segmentation software (AMIRA v. 4.1, Mercury Computer Systems Inc*.* USA). Neck vertebral geometries were generated from additional micro-CT scans (specimen YPM 9194—University of Texas, Austin, USA). These three-dimensional geometries were imported into ADAMS multibody analysis software (MSC Software Corp*.* USA) in preparation for an MDA. The skull had representative dimensions of length 68 mm, width 56 mm and height 35 mm. The total volume of the skull (including the bone and the sutures—as represented in figure 1) was approximately 10 160 mm^3^. Within ADAMS, detailed muscle anatomy was incorporated onto the geometries, and accurate jaw joint and tooth contact surfaces were specified. Where the neck met the skull a spherical joint was assigned that permitted the skull to rotate freely about all axes while constraining translational movements. The major adductor (jaw closing), depressor (jaw opening) and neck musculature were included, with each muscle group split into several sections and defined over the anatomical origin and insertions areas on the skull and lower jaws, respectively [57,60,62] (figure 2). To permit biting, a food bolus was modelled that could be located at any position along the jaw, and a specially developed motion technique, named dynamic geometric optimization [62], was used to simulate typical feeding movements. Simply, the line of action of each muscle is used to determine its level of activity during jaw movements. This motion technique, along with the muscle forces and biting performance, has been described and validated elsewhere [58,59,62].

Fifteen biting simulations were performed, including eight unilateral bites, five bilateral bites and two ripping bites (figure 2, as in [36]). During all bites, the adductor muscles were fully activated to ensure peak bite forces were generated. The ripping bites aimed to pull the head dorsally to the left, and dorsally to the right while biting down onto a fixed food bolus. This caused neck muscle forces to reach their maximum magnitudes. In all simulations, the lower jaws opened from a closed position to allow the food bolus to locate unobstructed at a specified tooth location. The lower jaws then closed to contact the food, upon which forces within all adductor muscle groups were ramped up until they reached their peak magnitudes. The MDA outputs muscle force location, direction and magnitude; joint contact location, direction and magnitude; and bite contact location, direction and magnitude for each biting simulation.

### 2.2. Finite-element analysis

The same CT dataset used to construct the MDA model was used to incorporate sutures into the skull. Sutures were integrated as a separate material by carefully tracing the gaps between the skull bone facets on the individual micro-CT slice images, so that all individual skull bones were completely isolated from one another (i.e. fully separated by the sutural soft tissue). This approach guaranteed that all sutures were represented in their entirety so that for loads to pass from one bone to another in the model it would have to pass through the sutural soft tissue material (three-dimensional model with sutures shown in figure 1).

The model was converted into a tetrahedral mesh consisting of 395 822 elements, constructed from solid (10 node) higher order elements. From these elements, 291 920 were assigned as bone and 103 902 were assigned as sutural soft tissue. Sensitivity studies (N. Curtis 2010, unpublished data) demonstrated that these were sufficient numbers of elements to accurately predict the strain through the model. Two set-ups were analysed: one representing fused sutures where the sutural soft tissue material was given the same material properties as bone (Young's modulus and Poisson's ratio of 17 and 0.3 GPa, respectively); and another representing patent sutures where the sutural soft tissue material was given a Young's modulus of 10 MPa and a Poisson's ratio of 0.3 MPa. All material properties were consistent with direct measurements and were within the ranges applied by other researchers [24,36–38,44,63–66].

A series of 15 FEAs was carried out on both the fused and patent suture models, with all muscle, joint and bite force locations, directions and magnitudes imported directly from the MDA simulations. Although theoretically all forces within the system should be in equilibrium, owing to the large number of individual forces, even small variations from the exact MDA locations of these applied forces would cause instability within the FEAs (i.e. there would be unconstrained full body motion of the model). Therefore, to ensure a stable FE solution, fixed constraints were included in the model. The locations of these constraints were taken at the joint and bite contacts as defined by the MDA (i.e. neck joint, jaw joints and bite point). One node at the neck location (occipital condyle) was constrained in the medial–lateral and anterior–posterior directions (*x*- and *z*-axes), one node at each jaw joint and bite point was constrained in the vertical direction (*y*-axis). These constraints were considered minimal, and restricted full body motion but not deformations of the skull. For example, the neck, bite and joint contact locations could all deform with respect to each other, and both jaw joint contact locations could deform relative to each other. After the FE solutions were complete, von Mises strains of all bone elements (291 920 elements) in the model were stored in element tables. A previous study carried out by Curtis *et al*. [36] showed von Mises strains to be a good indicator of bone performance. Investigating the strain in the sutural soft tissue material is beyond the scope of this study.

## 3. Results

### 3.1. Multibody dynamics analysis

The MDA simulations were similar to those carried out in a previous study, where more detailed results are presented [36]. From the MDA, muscle force locations, orientations and magnitudes; joint force locations, orientations and magnitudes; and bite force locations, orientations and magnitudes were predicted for 15 separate biting simulations (figure 2). Table 1 summarizes peak bite forces and joint forces predicted from the MDA. All predicted muscle, joint and bite forces were exported for use in the FEAs.

**Table 1.** Bite forces and jaw joint forces predicted by the MDA. Total forces are shown for bilateral bites; therefore, the force on each side of the skull is approximately half that presented. Working refers to the force on the same side as the bite occurs, while balancing refers to the opposite side to which biting occurs. See figure 2 for explanation of bite locations.

bite type	bite location	bite force (*N*)	working joint force (*N*)	balancing joint force (*N*)
bilateral	B1	121	540	—
	B2	150	524	—
	B3	165	510	—
	B4	185	490	—
	B5	214	462	—
unilateral	U2	150	249	276
	U3	166	232	276
	U4	187	212	277
	U5	216	183	278

### 3.2. Finite-element analysis

**Figure 3.**
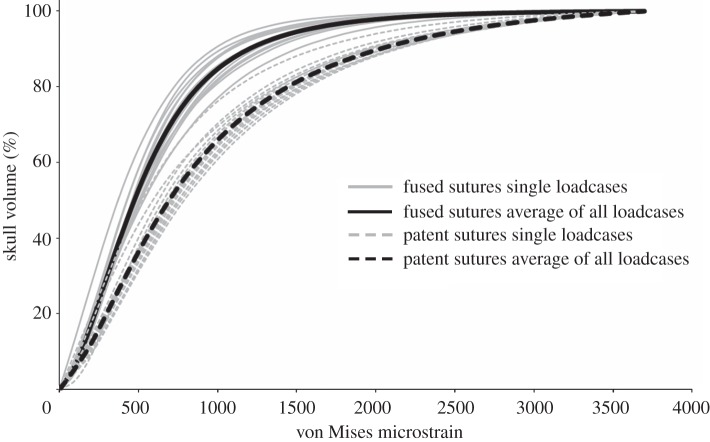
Cumulative strain plots showing the % volume of the skull (bone only not sutural soft tissue) at specific von Mises strain levels. Plots of all 15 individual loadcases along with the average of all loadcases are presented for both the fused and patent suture states.

**Figure 4.**
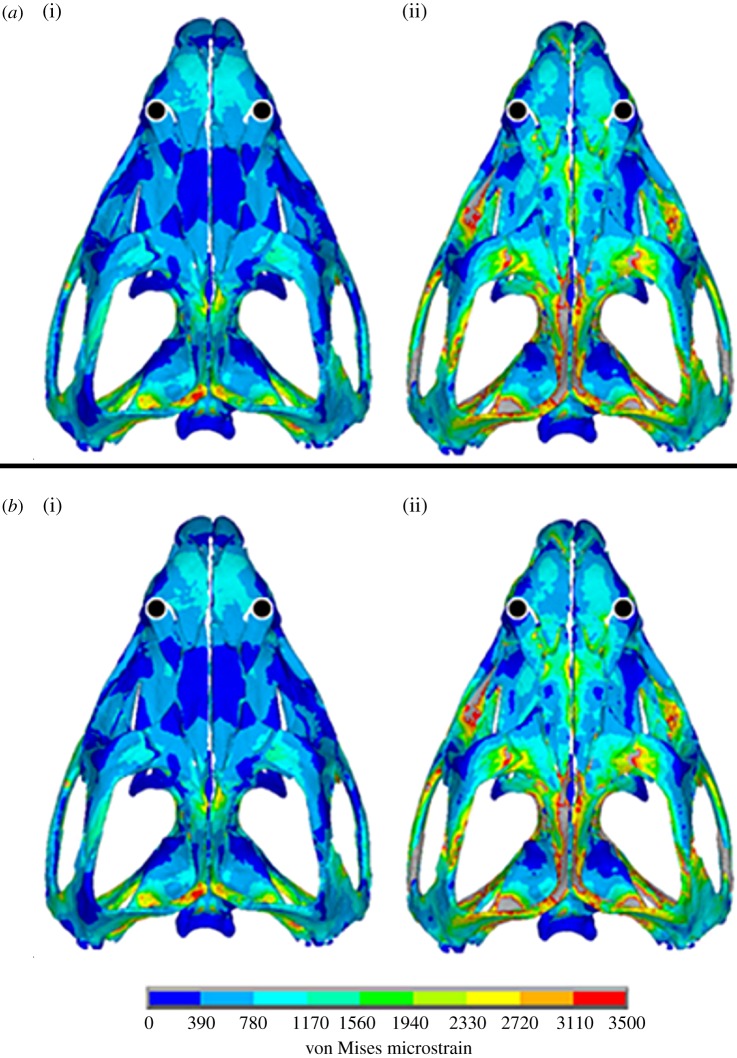
Sample von Mises strain distribution plots with (i) fused and (ii) patent suture states resulting from (*a*) an anterior bilateral bite and (*b*) a posterior unilateral bite. Black circle represents bite location. Suture material is not visible in these images.

Thirty separate FEAs were carried out on the same skull, 15 with sutures modelled as fused and 15 with sutures modelled as patent. In analyses where sutures were modelled as fused, individual bites generated areas of both high and low strain throughout the skull, with higher strains concentrated around muscle attachments and bite points. In such cases, 53 per cent of the skull volume was at strain levels of less than 500 microstrain (figure 3). When sutures were modelled as patent, it was immediately obvious that strains in some of the low-level strain regions were elevated (figure 4), with only 37 per cent of the skull volume at strains of under 500 microstrain (figure 3). This was noted across all 15 separate bites, with overall mean element strain (i.e. the average strain value of each element) throughout the skull increasing from approximately 655 microstrain (fused sutures) to approximately 1226 microstrain (patent sutures). The percentage volume of bone within the skull at specific strain magnitudes was similar for all individual loading cases with each suture state (i.e. fused or patent), but the percentage of bone at lower strain levels was reduced considerably when sutures were patent (figure 3).

## 4. Discussion

The adult skull of the New Zealand reptile *Sphenodon* contains many patent sutures [3], making it an ideal subject for an investigation of the impact of these sutures on overall skull performance. We used a combination of MDA and FEA to load the skull and assess the impact of patent sutures on skull strains in *Sphenodon*. As with all computer modelling investigations, there are some approximations that could impact on the model performance. In our experience, their impact is probably small in relation to the effects we observe, but it is important to be explicit about potential modelling limitations. All sutures were carefully and accurately positioned throughout the skull, but to allow appropriate meshing within the FE model the sutures needed to be enlarged and simplified. The non-enlarged sutures were approximately 0.35 mm wide, whereas the enlarged suture width was approximately 0.5 mm, but this did vary slightly throughout the skull. Enlarging the sutures may have reduced the constraining properties of the sutures and their relative deformations may be greater than found in nature. Another approximation concerns the material properties of the bone and sutural soft tissue material. Both were represented as isotropic and homogeneous structures, which in reality is not the case. Although these approximations will have some effect on strains generated, they would not be expected to alter either the general strain patterns or the differences in magnitude between the fused and patent models. As such, the findings and conclusions of this investigation would not be affected.

**Figure 5.**
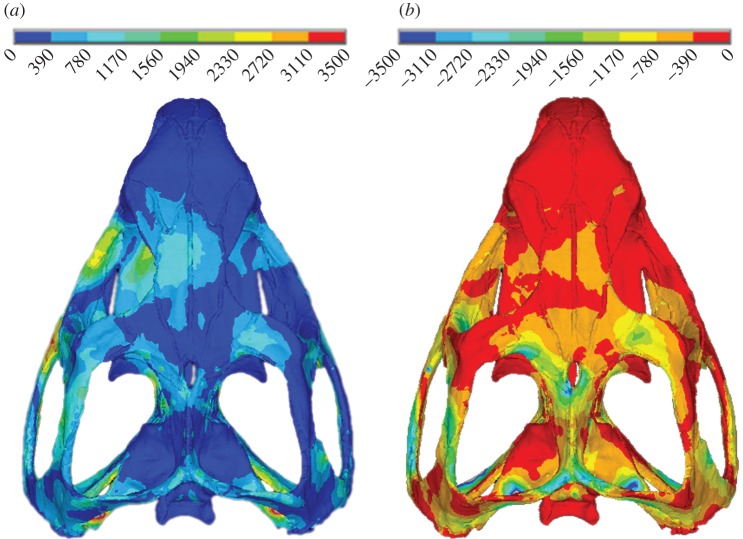
Example (*a*) first and (*b*) third principal strain plots for a single loadcase. Values in microstrain.

Our findings falsify hypotheses 1 and 2 (outlined in §1) in that the presence of patent sutures clearly impacts on stress and strain distributions by raising strains in certain skull regions. Thus, our experiment indicates that patent sutures lead to a more consistent higher strain magnitude over the skull, substantially limiting low-strain regions when compared with a fused suture model. While increasing bone strains may seem counterproductive, it could be an important consequence of patent sutures. Bone strain is thought to be the stimulus for bone modelling/remodelling, and if strains are too low or too high, bone will be removed or deposited accordingly [67–71]. It is therefore important for bone strains to be within an *equilibrium window* of strain. When sutures were modelled as fused within our FE simulations large regions of the skull experienced very low-strain magnitudes during biting. Identifying exact bone remodelling strain magnitudes is problematic and thus we cannot say categorically that our predicted strains with patent sutures would reduce the incidence of bone resorption; however, we are confident that our predicted peak strains are consistent with those recorded *in vivo* and *in vitro*. Peak (principal) strain magnitudes of between 900 and 5200 microstrain have been reported in bone during forceful loading [72–75], including 2000–3000 microstrain in a pig skull [30,76]. We noted peak tensile and compressive strains of approximately 2000–3000 microstrain in our study (figure 5).

Consistent with hypothesis 3, introducing anatomically accurate patent sutures into our model raised the strains in the very low-strain regions of the skull, generating a more widely distributed, high level of strain (figure 4). Thus, patent sutures offer two potential benefits. The first is a reduction of gross strain gradients (i.e. large regions of the skull at low strains and other regions at high strains), which could indicate a reduction of bending or twisting in the skull. Bone is more likely to fail under tensile strains, and these occur most often when bone is under bending or torsion [77]. However, it should be noted that strain gradients could be related to deformation regimens other than bending or twisting. The second potential benefit is that during each individual bite, irrespective of location or type, the general distribution of strain is similar. This reduces the chance of an area of the skull being under loaded. In figure 4, localized red *hotspots* are evident on the patent suture model, which indicate areas of high strain. The majority of these *hotspots* appear at locations where the bone in the model is very thin adjacent to the sutures. These *hotspots* are likely to be a result of the modelling limitations discussed earlier, whereby some sutures were expanded slightly so that they could be modelled with a sufficient number of reasonably shaped elements.

During growth and development, bone adapts more readily than at any other time in the animal's life. Low strains at this stage could result in under-developed/under-ossified bony structures, and the careful modulation of skull strains ensures the careful modulation of skull growth. Generating higher levels of strain throughout the adult skull is also important, and may be why some sutures remain patent even when the skull has stopped growing. There are several reasons why an animal may not bite at all points along the jaw at equal frequency: animals often have preferential sides of the jaw on which they bite more frequently [78,79], and different foods may require differential use of the jaw for their capture and breakdown. Additionally, the generally high levels of strain throughout the skull could render it more resilient in the face of pathologies that affect biting; ensuring bone is not lost or weakened due to short-term functional impairment. We have shown in *Sphenodon* that without patent sutures in the cranium the skull bones would only experience low-level strains, which could reduce their resistance to fracture.

These findings agree with previous studies on reptiles that suggest patent sutures modify strains substantially throughout the skull during biting [24]. However, the results are at variance with experimental and modelling studies on mammals that suggest some regions of the mammalian skull experience only very low strains during mastication [31,80]. Similarly, computer models of mammal skulls with some patent sutures do not differ significantly from skulls with no (or completely fused) sutures [52]. However, further discussion must await the analysis of a comparable mammalian skull with a full complement of patent sutures.

We hypothesize that most of the bone in the space-frame-type structure of the *Sphenodon* cranium is loaded principally through feeding activities and neck musculature, where the forces are intermittent but relatively high. Although equivalent biting and neck loads will also be experienced by a large part of the stiffened-shell-type cranium of mammals, other areas may experience lower magnitude but higher frequency loads. It may be these high-frequency–low-magnitude loads that help maintain bone in the low-strain areas in mammal skulls [71].

In conclusion, the results of this analysis reveal for the first time that patent sutures help reduce the number of areas of low-level strain throughout the reptile skull, leading to a more predictable and widely distributed high level of strain during every bite. This has important implications with respect to bone growth and remodelling in both juvenile and adult skulls, ensuring that bone grows (and is maintained) normally and optimally.
